# Simulation and Feedback in Health Education: A Mixed Methods Study Comparing Three Simulation Modalities

**DOI:** 10.3390/pharmacy6020041

**Published:** 2018-05-03

**Authors:** Lauren Tait, Kenneth Lee, Rohan Rasiah, Joyce M. Cooper, Tristan Ling, Benjamin Geelan, Ivan Bindoff

**Affiliations:** 1 Pharmacy Department, Townsville Hospital, Douglas 4814, Australia; lauren.tait@health.qld.gov.au; 2 Division of Pharmacy, University of Western Australia, Perth 6009, Australia; kenneth.lee@uwa.edu.au; 3 Western Australian Centre for Rural Health, University of Western Australia, Geraldton 6530, Australia; rohan.rasiah@uwa.edu.au; 4 School of Biomedical Sciences and Pharmacy, University of Newcastle, Newcastle 2308, Australia; Joyce.Cooper@newcastle.edu.au; 5 Department of Pharmacy, University of Tasmania, Hobart 7001, Australia; Tristan.Ling@utas.edu.au (T.L.); Benjamin.Geelan@utas.edu.au (B.G.)

**Keywords:** pharmacy education, simulation, scenarios, computer, virtual, virtual patient, standardized patient, actor, paper-based

## Abstract

**Background**. There are numerous approaches to simulating a patient encounter in pharmacy education. However, little direct comparison between these approaches has been undertaken. Our objective was to investigate student experiences, satisfaction, and feedback preferences between three scenario simulation modalities (paper-, actor-, and computer-based). **Methods**. We conducted a mixed methods study with randomized cross-over of simulation modalities on final-year Australian graduate-entry Master of Pharmacy students. Participants completed case-based scenarios within each of three simulation modalities, with feedback provided at the completion of each scenario in a format corresponding to each simulation modality. A post-simulation questionnaire collected qualitative and quantitative responses pertaining to participant satisfaction, experiences, and feedback preferences. **Results**. Participants reported similar levels satisfaction across all three modalities. However, each modality resulted in unique positive and negative experiences, such as student disengagement with paper-based scenarios. **Conclusion**. Importantly, the themes of guidance and opportunity for peer discussion underlie the best forms of feedback for students. The provision of feedback following simulation should be carefully considered and delivered, with all three simulation modalities producing both positive and negative experiences in regard to their feedback format.

## 1. Introduction

With the limited and variable availability of clinical placements [1,2], simulation-based education has increasingly become an adjunct approach in the comprehensive clinical training of students across medical, nursing, pharmacy and allied health disciplines [2,3]. While clinical placements remain a key component in facilitating the development of clinical expertise and competence, there is evidence to suggest that simulation-based education is suitable as a partial substitute and is an effective adjunct to clinical placements [4]. A recent systematic review about the use of simulation found that the substitution of clinical placement with simulation-based training did not appear to have a significant impact on overall clinical competence [5]. Similar to clinical placements, simulated activities can incorporate various practice elements within a single activity and can encourage students to reflect on their performance [1,6]. In addition, simulation can provide a safe and ethical learning environment, allowing students to learn at their own pace, with the ability to repeat clinical scenarios to further assist with knowledge consolidation and learning [1].

There are, however, a multitude of approaches to simulation, utilizing a variety of modalities. Broadly speaking, simulated activities can range from high to low fidelity, depending on the extent to which they reproduce real-world conditions [1]. The choice of modality depends upon the desired complexity of the activity and the intended learning outcomes. Common simulation modalities include paper-based, computer-based, human actor-based (simulated patient/role play), or mannequin-based [1]. While several approaches to simulation have been used successfully, there is a paucity of research directly comparing the different simulation modalities. Previous studies have commonly investigated the effects of simulation-based education on areas such as clinical competence, knowledge acquisition, and self-confidence [2,4,5,6,7]. Despite the current pedagogical focus on student-centered learning [8], there appears to be a paucity of research that has qualitatively explored students’ experiences across different simulation modalities. 

In the design of any simulation-based scenario, it is important that the method of feedback and discussion is considered and provided post-simulation. Feedback has been identified as a key mechanism for facilitating reflection, knowledge consolidation, and learning within education generally [9], as well as more broadly within simulation-based educational research, where the need for feedback or ‘debriefing’ is well established [10]. However, while the format and provision of feedback varies across educational media [10], students’ preferences for and appreciation of different formats of feedback have not been comparatively explored in the context of simulation-based education.

The present study’s objectives are thus to explore students’ (1) experiences with using paper-based, simulated patient (actor-based/role play), and computer-based simulations; (2) assessment of the satisfaction of completing clinical scenarios via the aforementioned modalities, and (3) preferences for the delivery of feedback provided post-simulation. Through exploration of the aforementioned objectives, the present study aims to identify key characteristics for designing simulated activities and feedback provision.

## 2. Materials and Methods 

This study was approved by the University of Newcastle Human Research Ethics Committee.

Academic teaching staff at the University of Newcastle (UON), Australia, sent a group email invitation to eligible participants to voluntarily participate in the present study. Eligible participants were students enrolled in a compulsory final year unit of a two-year postgraduate Master of Pharmacy coursework program at the UON. There was a total of 65 students eligible for participation; these students were selected as they have undergone sufficient training to possess the necessary clinical knowledge and communication skills required to complete the simulated scenarios. 

### 2.1. Study Design

**Figure 1:**
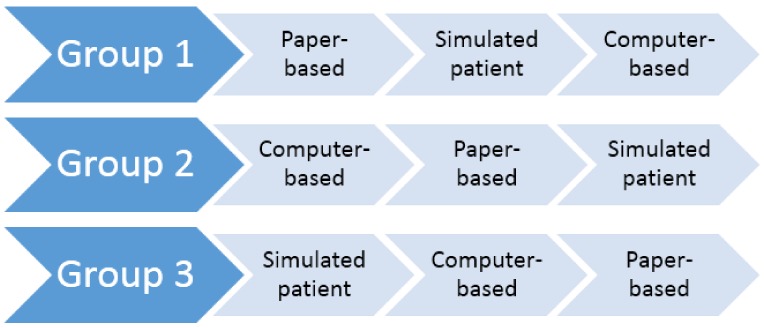
Schematic of the Group Allocations.

Participants were asked to complete three clinical scenarios consecutively, with one case-based scenario for each simulation modality (paper-based, computer-based, and simulated patient). A time frame of one hour was allocated to each of the simulated scenarios. The three scenarios were as follows: a scenario about a patient presenting to a community pharmacy with symptoms of gastrointestinal esophageal reflux disease (GERD), a patient with symptoms of angina, and a patient with symptoms of constipation. In each scenario, participants were required to undertake the role of a community pharmacist. Specifically, participants had to demonstrate knowledge of medical and medication history taking, identify the nature of the presenting complaint, and demonstrate knowledge of appropriate management options with either patient counselling or referral to an appropriate health professional. Educationally equivalent versions of each scenario were developed for each of the three simulation modalities. To mitigate a potential order bias in the presentation of the three simulation modalities, participants were randomized into three groups (Figure 1).

For each case-based scenario, the students were tasked with determining the condition of the patient and recommending the most appropriate course of action:

- The paper-based simulation was designed as a series of written questions whereby participants had to write the relevant questions they needed to ask the patient, the information they needed to provide the patient, and recommended courses of action.
- The computer-based simulation was designed as a three-dimensional computer game whereby participants could interact with the patient by selecting relevant text options (questions, advice, and actions) from a list of available text options; this computer simulation has been described in more detail in previous studies [11,12].
- The simulated patient (actor-based) simulation was designed as a role-playing exercise whereby actors played the role of a patient presenting to the pharmacy. These actors were given a standard backstory as a guide to follow.

On completion of each clinical scenario, participants were provided with feedback. The form of feedback differed according to the simulation modality.

- For the paper-based simulation, feedback was delivered immediately after scenario completion in the form of model answers to the written questions, accompanied by a small group discussion with other participants in the same group allocation. A practicing pharmacist, who was employed as a lecturer at UON advised the most appropriate course of action for the scenario and facilitated small group discussion.
- For the computer-based simulation, feedback was delivered automatically immediately after scenario completion as a detailed scorecard within the game. Specifically, an itemized list of the participant’s chosen text responses was presented alongside relevant feedback for each response, associated points for each response, as well as a total score.
- For the simulated patient simulation, a video recording of the participant’s role-play was provided to the participant, along with their overall score and feedback as judged by experienced pharmacists using a marking guide; this feedback was provided to the participant on the following day.

### 2.2. Data Collection

A series of three post-simulation, paper-based questionnaires were developed to capture demographics, explore participants’ experiences with each of the three simulation modalities, their satisfaction with completing clinical scenarios using each of the three modalities, and their experiences with the different forms of feedback associated with each of the three simulation modalities. In order to capture a rich understanding of participants’ experiences, both qualitative and quantitative data were collected for the experience questionnaires. Qualitative feedback was also gathered in free-form comments on this same questionnaire. The questionnaire can be seen in Appendix A.

### 2.3. Data Analysis

Quantitative data was analyzed using descriptive statistics; performed in SPSS version 23. 

Qualitative data from the paper-based questionnaires were transcribed into electronic format, and analyzed using a method based on Framework Method [13] of analysis, which is designed to elicit manifest- and latent-level themes from the data [14,15], with data management assistance from QSR NVivo version 11. Although elsewhere described in greater detail [13], the Framework Method follows common approaches of qualitative data analysis with the researcher (1) transcribing raw data into electronic text format; (2) familiarizing themselves with the data; (3) developing a working analytical framework; (4) applying and refining the analytical framework; and (5) identifying and making sense of the themes. However, the Framework Method adds to common qualitative data analysis processes by charting categorized data and presenting it in a matrix, which assists with the identification of themes.

In the present study, the analytical framework was firstly developed inductively by coding and categorizing data from the first few participant responses, and then it was continually refined as it was applied to the coding and categorization of remaining participants’ responses. The matrix was presented, with simulation modalities as columns, and participants’ categorized responses as rows. Viewing the categorized data along the columns facilitates identification of themes pertaining to participants’ experiences with each simulation modality and the associated form of feedback (manifest-level themes). Viewing categorized data along the rows allows identification of underlying patterns (latent-level themes) for participants’ experiences with the use of simulation and their feedback experiences.

To assure credibility and trustworthiness in the conduct of qualitative data analysis [16], all codes, categories, and themes were initially identified by a member of the research team with experience in qualitative research (KL), and then verified by another member of the research team (IB), with assistance from coding memos. The use of memos, along with constantly checking each code, category, and theme against the transcribed data collectively served as a means to ensure there was no drift in the coding, categorizing, and theme identification processes; this assured dependability in the data interpretation [16].

## 3. Results

Of the 65 students invited and eligible to participate in this study, 21 agreed to participate (32.31%) with 20 participants successfully completing the present study (95.24%). 

The following sections provide a more detailed summary of results. Owing to the small sample size and the greater potential for outliers to greatly skew means, median values have been included for comparison, where relevant.

### 3.1. Demographics

Table 1 summarizes the participants’ demographic characteristics. Additionally, when asked about their experience with playing computer games on a four-point Likert-type scale, 75% (15/20) indicated that they ‘never’ or ‘rarely’ play computer games, while the remainder indicated that they play computer games ‘sometimes’ or ‘often’.

### 3.2. Simulation Experience, Feedback Provision, and Overall Satisfaction

#### Descriptive Statistics

Participants were asked a series of questions on a five-point Likert-type scale (Appendix A) to ascertain their satisfaction with each clinical scenario, the use of each simulation modality for the clinical scenarios, and the use of each simulation modality for acquiring clinical knowledge and communication skills. Overall, participants appeared to be similarly satisfied across the three modalities (Table 2). Of note, participants appeared less satisfied with the computer-based simulation activity for Scenario Three (constipation). Interestingly, this scenario was the only one where the patient (actor-based) modality had the highest level of satisfaction, being the lowest rating in both other modalities. 

Table 3 summarizes participants’ quantitative responses to questions about feedback provision. Despite having the lowest satisfaction rating for two scenarios, participants rated the feedback from the patient (actor-based) modality as the highest for all three scenarios, indicating that participants found this form of feedback most useful.

### 3.3. Manifest-Level Themes

Table 4 summarizes the manifest-level themes for participants’ simulation experiences and associated feedback provision for each simulation modality. When comparisons were made between participants who reported currently working in pharmacy versus participants who were not currently working in a pharmacy setting, the same identified themes were present in both groups, with one notable difference: the theme of ‘low engagement’ for the paper-based simulation was only identified for participants who reported currently working in pharmacy. When comparisons were made between participants who reported ‘never’ or ‘rarely’ versus participants who reported ‘sometimes’ or ‘often’ playing computer games, the technological issues identified for the computer-based simulation prevailed across groups.

While overall satisfaction and satisfaction with the use of the various simulation modalities for completing clinical scenarios were not directly explored qualitatively, participants’ qualitative responses to other questions commonly indicated satisfaction:

“Different and fun way of learning” [computer-based simulation]

“Great learning experience. Shows real life scenarios that can happen within a pharmacy” [simulated patient simulation]

“Interactive component was the best as you got to discuss answers and hear other people’s opinions” [feedback for the paper-based simulation]

One participant even recommended the use of simulation as a tool for learning for other students:

“I think it was a good learning modality that should be given to first year students as it will allow students to practice communicating with a customer before being exposed to real life situations” [computer-based simulation]

### 3.4. Latent-Level Themes

Underlying participants’ simulation experiences, feedback provision, and overall satisfaction across modalities, two central themes were identified: the desire to be engaged throughout the learning activity, and the desire for guided learning and knowledge consolidation throughout the learning activity. Participants expressed both positive and negative sentiments relating to ‘engagement’:

“Fun way to put pharmacy practice into reality” [computer-based simulation]

“Boring, does not simulate true experiences” [paper-based simulation]

However, while the manifest-level theme of low engagement was identified for the paper-based simulation, comments relating to the feedback component of the simulation suggest that participants enjoyed the feedback component (Table 4). 

In regards to guided learning and knowledge consolidation, participants commonly expressed a desire for guidance through peer learning. For example:

“The feedback was given straight after the tutorial. Was really good to go through the answers at the end with everyone because you can learn from your peers” [feedback for paper-based simulation]

Where there was a lack of peer interaction, negative sentiments were expressed towards the simulation modality/form of feedback (Table 4).

Despite the computer-based simulation reportedly providing detailed feedback, the perceived lack of guidance was identified:

“Feedback is very structured and clear, but we may need more detailed feedback on when to ask some particular questions” [computer-based simulation]

Reflection was expressed as another way to guide learning and consolidate knowledge. However, spatial and temporal factors appear noteworthy when considering reflection as a way to guide learning and knowledge consolidation:

“Feedback was given straight away which is good to review your strengths and weaknesses” [feedback for computer-based simulation]

“This is the best way to give feedback but may not be the fastest way due to its own nature” [feedback for simulated patient simulation]

Additionally, guidance is important throughout the simulation:

“Little bit complicated to navigate initially but was good after I understood how to work it” [computer-based simulation]

“I wasn’t aware the patient would just walk in and the scenario would start, was a little off putting and forgot to introduce myself” [simulated patient simulation]

## 4. Discussion

### 4.1. Principal Findings

Participants reported that they were highly satisfied overall with the use of simulation for clinical-based learning across all three modalities; this finding was evident in participants’ quantitative responses for both their overall satisfaction and feedback perceptions for each modality (Table 2 and Table 3 respectively) but was also identifiable within their responses to qualitative questions. Satisfaction with the use of simulation supports previous studies that have suggested the utility of simulation as a supplementary learning tool for the development of clinical knowledge and skills [3,4,5]. Despite overall high levels of satisfaction, the present study highlights the need to consider and address potential dampeners to a positive simulation experience, such as technology-related issues (Table 4). 

The present study provides insight into a variety of positive and negative aspects of three common simulation modalities, from the perspective of students. For example, the paper-based simulation was perceivably less interesting than the other modalities (Table 4). However, despite the high level of realism perceived by participants for the simulated patient simulation, psychosocial factors such as the potential for the modality to heighten/induce anxiety in students should be recognized (Table 4). Additionally, as partly echoed in the literature, the choice of modality should be considered within the broader pedagogical approach [6] and the intended learning outcomes. For example, the simulated patient simulation was reported by participants as a suitable modality for developing and consolidating communication skills (Table 4). 

In regards to the provision of feedback, participants in the present study indicated a desire for peer discussion, as offered by the post-paper based simulation activities (Table 4). While the level of detail provided in the score card of the computer-based simulation was reportedly comprehensive (Table 4), participants reported that the feedback needed to offer greater guidance for future courses of action. Similarly, the use of a video recording as feedback for the simulated patient simulation was positively received by participants. However, the time delay reportedly impacted on the value of the feedback and its utility for reflection and subsequent knowledge consolidation.

Underlying the manifest-level themes identified for each modality and form of feedback were the latent-level themes of engagement and guidance. Although the paper-based simulation appeared less engaging, the associated feedback appeared to engage participants. Given that engagement has been identified in previous studies as an important factor in student learning [17,18], the concept of engagement should be considered throughout the implementation and design of a simulation activity, including post-simulation feedback. In addition, the theme of guidance throughout the learning activity, both during the simulation and particularly for feedback provision, highlights the importance of ensuring students know what they need to do with the information presented. 

### 4.2. Limitations

While the sample size used in the current study is not uncommon for qualitative studies, as this is a mixed method study, the sample size and the selection of only pharmacy students from a single institution should be acknowledged as key limitations to the generalizability of the study’s quantitative findings. Future studies should consider utilizing a larger sample size to enable inferential statistical analyses, as well as selecting students from other institutions and health disciplines. Nevertheless, the collection of qualitative alongside quantitative data allowed for a rich understanding of a range of students’ experiences with simulation and feedback provision. 

Additionally, the randomization design of the ordering of simulation modalities did not allow for direct statistical comparison of the same scenario across modalities. This randomization design was chosen in light of the anticipated limited sample size. 

## 5. Conclusions 

The present study highlights a number of positive and negative aspects of three common simulation modalities, as well as the form of feedback provision. Despite some unique differences, underlying commonalities suggest that engagement and guidance throughout a simulation activity should be considered: both during a simulation, and post-simulation feedback.

## Figures and Tables

**Table 1:** Demographic Characteristics.

Sex	n (%)
Male	3 (15.0)
Female	17 (85.0)
Age Range (years)	
22–25	11 (55.0)
26–29	4 (20.0)
30+	5 (25.0)
Currently Working in a Community Pharmacy	
Yes	11 (55.0)
No	9 (45.0)

**Table 2:** Participant Satisfaction with Each Scenario and Simulation Modality.

Satisfaction Items	Scenario One: Reflux			Scenario Two: Angina			Scenario Three: Constipation		
	Paper	Computer	Patient	Paper	Computer	Patient	Paper	Computer	Patient
	Median (Mean)	Median (Mean)	Median (Mean)	Median (Mean)	Median (Mean)	Median (Mean)	Median (Mean)	Median (Mean)	Median (Mean)
**Percentage of total maximum rating ^1^:**	**91.8% (77.8%)**	**90.0% (78.7%)**	**81.8% (76.9%)**	**90.9% (78.4%)**	**80.0% (73.1%)**	**80.0% (75.8%)**	**76.4% (70.0%)**	**70.9% (60.9%)**	**91.8% (78.5%)**
The scenario was fun to work through	4.0 (4.0)	4.5 (4.5)	4.0 (4.0)	4.5 (4.0)	4.0 (4.3)	4.0 (4.1)	3.0 (3.4)	3.0 (3.3)	4.5 (4.2)
I was interested in the subject matter covered by the scenario	5.0 (4.6)	5.0 (4.8)	4.0 (4.3)	5.0 (4.5)	4.0 (4.1)	4.0 (4.3)	4.0 (4.0)	4.0 (4.0)	4.5 (4.3)
This was an engaging way to present to scenario	4.5 (3.8)	5.0 (4.7)	4.0 (4.4)	4.0 (3.8)	4.0 (4.1)	4.0 (4.3)	4.0 (3.6)	4.0 (3.9)	4.5 (4.3)
Working through the scenario helped me learn about the subject matter	5.0 (4.9)	4.5 (4.3)	4.0 (4.3)	4.0 (4.3)	4.0 (4.0)	4.0 (3.7)	4.0 (4.1)	3.0 (3.1)	4.0 (4.2)
I found the scenario easy to complete	5.0 (4.6)	4.0 (3.7)	3.0 (3.4)	4.0 (4.0)	4.0 (4.4)	4.0 (3.9)	4.0 (4.0)	3.0 (2.4)	4.0 (3.5)
I think this was a good way to learn about pharmacy practice issues	4.0 (4.3)	4.0 (4.3)	4.0 (4.4)	5.0 (4.7)	4.0 (4.1)	4.0 (4.3)	4.0 (3.9)	3.0 (3.2)	5.0 (4.7)
I think this was a good way to learn about taking a patient/medication/medical history	4.0 (4.0)	4.0 (4.0)	5.0 (4.6)	5.0 (4.7)	4.0 (3.7)	4.0 (4.4)	4.0 (3.9)	3.0 (3.0)	5.0 (4.7)
I think this was a good way to learn about counselling patients	5.0 (4.3)	4.0 (3.8)	5.0 (4.6)	3.5 (3.8)	4.0 (3.7)	4.0 (4.4)	3.0 (3.7)	4.0 (3.4)	5.0 (4.8)
I think this was a good way to learn about medication/health condition related problems	5.0 (4.3)	4.5 (4.5)	4.0 (4.0)	5.0 (4.8)	4.0 (3.9)	4.0 (4.0)	4.0 (4.0)	4.0 (3.6)	4.0 (3.8)
I would like to work through this scenario again using this learning modality	4.0 (4.0)	5.0 (4.7)	4.0 (4.3)	5.0 (4.5)	4.0 (3.9)	4.0 (4.3)	4.0 (3.9)	4.0 (3.6)	5.0 (4.7)
I would like to work through different scenarios using this learning modality	5.0 (4.7)	5.0 (4.7)	4.0 (4.4)	5.0 (4.7)	4.0 (4.0)	4.0 (4.3)	4.0 (3.7)	4.0 (3.9)	5.0 (4.7)

^1^ Percentage of total maximum rating calculated by dividing the average score of all questions by 5, and multiplying by 100.

**Table 3:** Participants’ Perceptions of the Feedback Provided in Each Scenario and Simulation Modality.

Feedback Items	Scenario One: Reflux			Scenario Two: Angina			Scenario Three: Constipation		
	Paper	Computer	Patient	Paper	Computer	Patient	Paper	Computer	Patient
	Median (Mean)	Median (Mean)	Median (Mean)	Median (Mean)	Median (Mean)	Median (Mean)	Median (Mean)	Median (Mean)	Median (Mean)
**Percentage of total maximum rating ^2^:**	**98.2% (86.0%)**	**84.5% (82.4%)**	**98.2% (92.2%)**	**95.5% (91.8%)**	**80.0% (80.4%)**	**100% (95.5%)**	**92.7% (85.6%)**	**72.7% (72.0%)**	**100% (97.5%)**
Feedback provided for this scenario was provided in a timely manner	5.0 (4.6)	5.0 (4.7)	5.0 (4.7)	5.0 (4.7)	4.0 (4.3)	5.0 (4.4)	5.0 (4.4)	5.0 (4.4)	5.0 (4.8)
Feedback provided from the learning modality was constructive and clarified what good performance is	5.0 (4.4)	4.5 (4.2)	5.0 (4.7)	5.0 (4.7)	4.0 (4.1)	5.0 (4.9)	4.0 (4.4)	4.0 (4.0)	5.0 (4.8)
Feedback provided allowed me to reflect on my performance	5.0 (4.6)	4.5 (4.3)	5.0 (4.7)	5.0 (5.0)	4.0 (4.1)	5.0 (4.9)	5.0 (4.4)	4.0 (4.0)	5.0 (5.0)
The feedback provided motivates my learning	5.0 (4.1)	4.5 (4.2)	5.0 (4.6)	5.0 (5.0)	4.0 (4.0)	5.0 (4.9)	5.0 (4.4)	4.0 (4.0)	5.0 (4.8)
The feedback provided allowed me to analyze my patient history taking skills	5.0 (4.4)	4.0 (4.2)	5.0 (4.7)	4.5 (4.5)	4.0 (4.4)	5.0 (4.9)	4.0 (4.1)	4.0 (3.7)	5.0 (4.8)
The feedback provided allowed me to self-analyze my clinical knowledge	5.0 (4.9)	4.0 (4.2)	5.0 (4.6)	4.5 (4.5)	4.0 (4.1)	5.0 (4.9)	5.0 (4.4)	4.0 (4.0)	5.0 (5.0)
Feedback provided allowed me to analyze/self-assess my patient counselling advice	5.0 (4.1)	4.0 (4.2)	5.0 (4.7)	5.0 (4.7)	4.0 (3.7)	5.0 (4.7)	5.0 (4.3)	4.0 (3.9)	5.0 (4.8)
Feedback provided allowed me to reflect on my communication skills	5.0 (4.0)	4.0 (4.0)	5.0 (4.7)	5.0 (4.7)	4.0 (3.7)	5.0 (4.9)	4.0 (4.1)	3.0 (3.3)	5.0 (4.8)
The feedback provided allowed me to reflect on my verbal communication skills	5.0 (3.7)	4.0 (3.8)	5.0 (4.4)	4.0 (3.8)	4.0 (3.9)	5.0 (4.9)	4.0 (4.0)	2.0 (2.4)	5.0 (5.0)
The feedback provided allowed me to reflect on my non-verbal communication skills	4.0 (4.1)	4.0 (3.5)	4.0 (4.3)	4.5 (4.2)	4.0 (3.9)	5.0 (4.4)	5.0 (4.3)	3.0 (2.9)	5.0 (4.8)
Feedback provided will alter how I approach future pharmacy practice issues	5.0 (4.4)	4.0 (4.0)	5.0 (4.6)	5.0 (4.7)	4.0 (4.0)	5.0 (4.7)	5.0 (4.3)	3.0 (3.0)	5.0 (5.0)

^2^ Percentage of total maximum rating calculated by dividing the average score of all questions by 5, and multiplying by 100.

**Table 4:** Manifest-Level Themes for Each Simulation Modality Illustrating Positive and Negative Aspects of Both Experience and Feedback.

Paper-Based Themes	Computer-Based Themes	Simulated Patient (Actor-Based) Themes
**Simulation Experience**	**Simulation Experience**	**Simulation Experience**
(+) Allowed for knowledge consolidation and learning (+) Self-directed (no time limit) (+) Non-confronting/safe environment (−) Low engagement (boring) (−) Low realism (−) Low interactivity (−) Lengthy activity (−) Low degree of difficulty (−) No communication skills practice	(+) Allowed for knowledge consolidation and learning (+) Self-directed (no time limit) (+) High engagement (fun) (+) Allowed for repetition of scenario (+) Non-confronting/safe environment (+) Innovative way of learning (+) High interactivity (+/−) Good degree of realism but limited response options (−) Technology-related/usability issues (−) Did not allow communication skills practice	(+) Allowed for knowledge consolidation and learning (+) Allowed for communication skills practice (+) High interactivity (+/−) Good degree of realism but patient emotions perhaps too positive (−) Feeling of anxiousness/nervousness (−) Time pressure (not self-directed)
**Feedback**	**Feedback**	**Feedback**
(+) Allowed for knowledge consolidation and learning (+) Facilitated reflective learning (+) High interactivity (+) Feedback facilitated by tutor (+) Peer discussion/learning (+) Immediacy of feedback (−) Perception of judgement from peers (−) Feedback too brief	(+) Immediacy of feedback (+) Facilitates reflective learning (+) Detailed feedback (−) No option for clarification/lacks guidance (−) Lacks peer discussion/learning	(+) Detailed feedback (+) Facilitated reflective learning (+) Observation of self as a good form of feedback (+) Personalized feedback (−) Lacks immediacy

(+) denotes positive sentiments, (−) denotes negative sentiments.

## Appendix A. Student Satisfaction

Please indicate your level of agreement with the following statements, by placing a tick in the appropriate box.

**1 = SD—Strongly Disagree**

**2 = D—Disagree**

**3 = N—Neutral**

**4 = A—Agree**

**5 = SA—Strongly Agree**

**Statement**	**SD 1**	**D 2**	**N 3**	**A 4**	**SA 5**
The scenario was fun to work through					
I was interested in the subject matter covered by the scenario					
This was an engaging way to present the scenario					
Working through the scenario helped me learn about the subject matter					
I found the scenario easy to complete					
I think this was a good way to learn about pharmacy practice issues					
I think that this was a good way to learn about taking a patient/medication/medical history					
I think this was a good way to learn about counselling patients					
I think this was a good way to learn about medication/health condition related problems					
I would like to work through this scenario again using this learning modality					
I would like to work through different scenarios using this learning modality					

(1) Please write any positive points you have about this modality of learning
  _________________________________________________________________________________________________________________________________________________________________________________________________________________________________
(2) Please write any negative points you have about this modality of learning
  _______________________________________________________________________________________________________________________________________________________________________________________________________________________________________________________________________________________________________________________________________________________________________________________
(3) Please provide any other comments in relation to this learning modality
  __________________________________________________________________________________________________________________________________________________________________________________________________________________________________________________________________________________________________________________________________________________________________________________________________________________________________________________________________
